# Oral Administration of Water Extract from *Euglena gracilis* Alters the Intestinal Microbiota and Prevents Lung Carcinoma Growth in Mice

**DOI:** 10.3390/nu14030678

**Published:** 2022-02-05

**Authors:** Deepa Upreti, Susumu Ishiguro, Nicole Robben, Ayaka Nakashima, Kengo Suzuki, Jeffrey Comer, Masaaki Tamura

**Affiliations:** 1 Department of Anatomy & Physiology, Kansas State University College of Veterinary Medicine, Manhattan, KS 66506, USA; deepa07@vet.k-state.edu (D.U.); isusumu@vet.k-state.edu (S.I.); ndrobben@vet.k-state.edu (N.R.); jeffcomer@ksu.edu (J.C.); 2 Euglena Co., Ltd., Minato-ku, Tokyo 108-0014, Japan; nakashima@euglena.jp (A.N.); suzuki@euglena.jp (K.S.)

**Keywords:** natural product, water extract from *Euglena gracilis*, lung cancer prevention, gut microbiota alteration, fecal microbiota transplantation

## Abstract

The antitumor effects of a partially purified water extract from *Euglena gracilis* (EWE) and EWE treated by boiling (bEWE) were evaluated using orthotopic lung cancer syngeneic mouse models with Lewis lung carcinoma (LLC) cells. Daily oral administration of either EWE or bEWE started three weeks prior to the inoculation of LLC cells significantly attenuated tumor growth as compared to the phosphate buffered saline (PBS) control, and the attenuation was further enhanced by bEWE. The intestinal microbiota compositions in both extract-treated groups were more diverse than that in the PBS group. Particularly, a decrease in the ratio of Firmicutes to Bacteroidetes and significant increases in *Akkermansia* and *Muribaculum* were observed in two types of EWE-treated groups. Fecal microbiota transplantation (FMT) using bEWE-treated mouse feces attenuated tumor growth to an extent equivalent to bEWE treatment, while tumor growth attenuation by bEWE was abolished by treatment with an antibiotic cocktail. These studies strongly suggest that daily oral administration of partially purified water extracts from *Euglena gracilis* attenuates lung carcinoma growth via the alteration of the intestinal microbiota.

## 1. Introduction

Lung cancer is a commonly diagnosed cancer type in both sexes and the leading cause of cancer-related deaths, comprising one in five cancer-related deaths worldwide [1]. Furthermore, lung cancer remains a leading cause of cancer-related death even among individuals who have never smoked [2,3]. Although many new strategies for cancer prevention and treatment are being implemented, lung cancer is predicted to cause the greatest number of deaths in 2021, in comparison to other cancer types [4]. In addition, many cancers tend to metastasize in the lungs [5], resulting in life-threatening conditions. Therefore, novel prevention and therapeutic strategies for lung cancer are urgently needed.

*Euglena gracilis* (*E. gracilis*) is a single-celled, flagellated, fast-proliferating microalga that is rich in nutrients [6]. This alga can be found in both fresh and saltwater and possesses characteristics of both plants and animals [7]. *E. gracilis* is used as a nutritional dietary supplement because it is rich in carbohydrates, amino acids, minerals, chlorophyll, and vitamins [8]. *E. gracilis* also serves as an excellent source of valuable substances such as paramylon (a water-insoluble granule or linear form of β-1,3-glucan), tocopherol, and carotenoids with pharmaceutical potentials [9]. *E. gracilis* with paramylon is known to have an immunomodulatory effect in both humans and animals, playing a role as an immunostimulant or an immunopotentiator [10,11]. Studies have revealed that *E. gracilis* has potential as a therapeutic due to its antimicrobial [12], antioxidant [13], anticancer [14], antiviral [15,16,17], and antihyperglycemic properties [18]. Furthermore, our previous study showed that the partially purified extract derived from *E. gracilis* prevents lung carcinoma growth in mice by stimulating host antitumor immunity through the attenuation of myeloid-derived suppressor cell (MDSC) populations [19].

The gut is colonized by complex bacterial populations, many of which are beneficial for the health to the host, including protection against pathogenic microbiota, production of vitamins, absorption of ions, etc. [20]. More importantly, the gut microbiota is essential for the development, training, and function of the immune system both locally and at distant sites [21,22]. Moreover, gut microbiota-derived metabolites, such as short-chain fatty acids (SCFA), can influence host immune responses [23]. Emerging evidence demonstrates vital crosstalk between the gut microbiota and the lung through a shared mucosal immune system termed the “gut–lung axis” [21], highlighting the impact of gut microbes in lung diseases such as asthma, chronic obstructive pulmonary disease, and lung cancer [24]. In fact, there is a significant difference in the gut bacterial composition of lung cancer patients in comparison to healthy controls [25]. These studies highlight the potential of manipulating the gut microbiota and/or metabolites as a therapeutic approach for lung diseases.

Our previous finding that the oral administration of partially purified extracts of *E. gracilis* prevents the growth of lung cancer in mice indicates a possible interaction along the gut–lung axis [19]. Another study reported that the components of *E. gracilis* stimulate the growth of gut microbes such as *Faecalibacterium* to improve digestive health by producing butyrate, an SCFA [26]. Here we examine the mechanism behind the prevention of lung carcinoma in mice upon daily administration of *E. gracilis* water extract. We find that the oral administration of *Euglena* water extract alters the gut microbiota and plays a role in the attenuation of lung carcinoma growth. Our findings indicate that water extract from *E. gracilis* could be used as a prebiotic for the prevention of lung cancer.

## 2. Materials and Methods

### 2.1. Animals

Wild-type female C57BL/6 mice were purchased from Charles River Laboratories International, Inc. and were housed in a clean facility under controlled conditions of temperature (20–26 °C), with 30–70% relative humidity and light (12:12 h light-dark cycles). All mice were held for a week to acclimatize before the treatment. All mice were housed humanely according to university, state, and federal guidelines (AAALAC) in the AAALAC-accredited animal resource facilities of the Kansas State University College of Veterinary Medicine. The condition of the mice was observed daily, and body weights were measured every three days. All experiments were carried out under approvals of the Kansas State University Institutional Animal Care and Use Committee (Protocol # 4346) and Institutional Biosafety Committee (Registration # 1317).

### 2.2. Preparation of Extracts from E. gracilis

Dried powder of *E. gracilis* was obtained from Euglena Co., Ltd. (Tokyo, Japan). Water extracts of *E. gracilis* were prepared by suspending this dried powder in sterile phosphate buffered saline (PBS) and incubating it at 37 °C for 30 min with periodic sonication for 30 s. The whole suspension was centrifuged at a low-speed of 34× *g* for 1 min. While the precipitate was separated and used to prepare paramylon water extract (PWE), the supernatant was used to prepare the *Euglena* water extract (EWE) and boiled *Euglena* water extract (bEWE). The precipitate was suspended in PBS, followed by centrifugation at 1240× *g* for 10 min. To obtain the substance denoted PWE, the precipitate derived from the first low speed centrifugation was boiled for 10 min, followed by a high-speed centrifugation at 11,405× *g* for 10 min. The resulting supernatant was used as the PWE. To obtain EWE and bEWE, the supernatant was centrifuged at 11,405× *g* for 10 min and then filtered by a 0.22 μm sterile disk filter (Midwest Scientific, Valley Park, MO, USA). The difference between EWE and bEWE is that a boiling process was carried out before the filtration step during the preparation of bEWE. The dry weight was calculated after drying a 300 μL aliquot at 55 °C for 48 h from which the dry weight of PBS was subtracted (0.125 mg dry powder/mL). All the partially purified water extracts from *E. gracilis* were stored at −20 °C until further use.

### 2.3. Cell Culture

The mouse Lewis lung carcinoma (LLC) cell line (CRL-1642) was purchased from American Type Culture Collection (ATCC, Manassas, VA, USA). Dulbecco’s Modified Eagle’s Medium (DMEM) was obtained from Mediatech, Inc. (Manassas, VA, USA). Fetal bovine serum (FBS) was obtained from Biowest (Riverside, MO, USA). The penicillin-streptomycin stock was obtained from Lonza Rockland, Inc. (Allendale, NJ, USA). The cells were cultured at 37 °C in a humidified air atmosphere containing 5% CO_2_. The cell line was authenticated by short tandem repeat (STR) DNA profiling. Both the cells were maintained in low passages (<15) for this study.

### 2.4. Treatment of Mice with Euglena Extracts via Drinking Water

**Figure 1:**
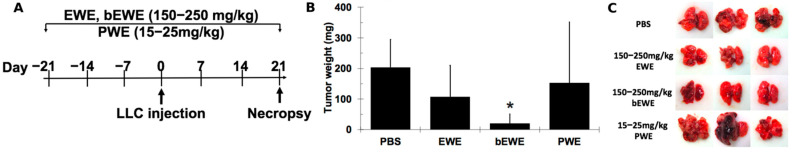
Pre-treatment with *Euglena* water extract (EWE) and boiled *Euglena* water extract (bEWE) attenuated growth of Lewis lung carcinoma (LLC) tumors in murine lungs. (**A**) Schematic illustration of the study design. (**B**) Average tumor weight in each treatment group; phosphate buffered saline (PBS) served as a control. (**C**) Macroscopic views of the lungs from LLC tumor-bearing mice in PBS or extract pretreatment groups. The scale bar in each picture represents 5 mm. Results are presented as mean ± SD (*n* = 5). *: *p* < 0.05 as compared to the PBS group. PWE: paramylon water extract.

The effect of oral administration of EWE and bEWE on lung cancer growth was evaluated using an orthotopic LLC cell syngeneic model in C57BL/6 mice (*n* = 4–5). Four treatment regimens were used: (1) PBS control, (2) 150–250 mg/kg/mouse/day EWE, (3) 150–250 mg/kg/mouse/day bEWE, (4) 15–25 mg/kg/mouse/day PWE (Figure 1A). The extracts were orally administered daily by drinking water three weeks before LLC cell inoculation via tail vein injection (2 × 10^6^ cells/mouse). Consumption of drinking water containing extracts was monitored by measuring the weight of the water pouches every three days. Based on the average water consumption (Figure S2), doses of each extract were adjusted to maintain the desired dosage. Each mouse was monitored daily for signs of illness. Three weeks after LLC cell injection, all mice were sacrificed by cervical dislocation following exposure to saturated CO_2_. The lung, spleen, both of the kidneys, liver, and intestine were collected to examine their weights and fixed in 10% formalin for histological analysis. The tumor weights in the lungs were calculated by subtracting the average weight of an age-matched normal mouse lung (142.3 ± 11.9, *n* = 5).

### 2.5. Fecal Sample Collection and 16S rRNA Amplicon Sequencing

Feces were collected from mice receiving each of the *Euglena* water extracts (EWE, bEWE, or PBS), at three time-points; Day 0 (before the treatment), Day 21 (before LLC cell injection), and Day 42 (before necropsy) and kept at −80 °C for later use. DNA was isolated from fecal pellets (200 mg) from the treated groups using the DNeasy PowerSoil Kit (Qiagen, Germantown, MD, USA). Genomic DNA samples were quantified using the Nanodrop system (Thermo Scientific, Waltham, MA, USA) and kept at −80 °C for further use.

The DNA samples were then processed for 16S rRNA sequencing by the Next Generation Sequencing Service of the Veterinary Diagnostic Lab (Kansas State University). The V3/V4 hypervariable region of the 16S rRNA gene was sequenced as specified by the manufacturer (Illumina, San Diego, CA, USA). Libraries were sequenced on an Illumina Miseq. Raw reads were trimmed for quality using FastQC v0.11.9 and analyzed using Mothur v1.44.1 [27,28,29]. The unique 16S reads, the output of Mothur (operational taxonomic units (OTUs)), were aligned to reference sequences from the SILVA rRNA database [30]. Near-identical sequences were merged using VSEARCH v2.15.1 [31].

### 2.6. Fecal Microbiota Transplantation

Fecal microbiota transplantation (FMT) was performed to determine the effect of the gut microbiota on the prevention of lung cancer in orthotopic LLC cell allografted mice. Four treatment regimens were performed: (1) PBS control, (2) 150–250 mg/kg of bEWE for 5 weeks prior to and 3 weeks after LLC cell injection, (3) FMT for 2 weeks prior to and 3 weeks after LLC cell injection, (4) 150–250 mg/kg of bEWE treatment throughout the experiment along with antibiotics (ABX) for 2 weeks prior to and 3 weeks after LLC cell injection.

After 3 weeks of bEWE administration via drinking water, feces were collected under sterile conditions and stored at −80 °C until further use for FMT. About 5–6 fecal pellets (about 100 mg) were thawed on ice and re-suspended in 1 mL Milli-Q water. The solution was vigorously mixed for 30 s by vortex and then centrifuged at 800× *g* for 3 min. About 500–600 μL of supernatant was collected and administered by oral gavage (100  μL/mouse/day) immediately after the collection [32]. Depletion of the gut microbiota was performed using a cocktail of ABX consisting of the following: ampicillin (1 g/L) (USBiological Life Sciences, Salem, MA, USA), kanamycin (1 g/L) (Thermo Scientific, Waltham, MA, USA), metronidazole (1 g/L) (Cayman Chemical Company, Ann Arbor, MI, USA), and vancomycin (0.5 g/L) (Cayman Chemical Company, Ann Arbor, MI, USA) as previously described [33,34]. ABX treatment was conducted out via oral gavage (100 μL/mouse/day).

### 2.7. Statistical Analysis

The diversity of microbial populations within samples (alpha diversity) was assessed using the Chao1 estimator and observed indices. Comparison of microbial communities between samples (beta diversity) was evaluated using Principal coordinate analysis plots (PCoA), based on Bray-Curtis distances which demonstrated the dissimilarity of microbiota between samples and treatments. This was compared using the nonparametric analysis of similarities (ANOSIM) test. Microbiota statistical analysis was carried out using MicrobiomeAnalyst software [35].

All values are expressed as the mean ± standard deviation of the mean. For all the experiments, statistical significance was assessed by an unpaired *t*-test and was conducted with multiple sample determinations. A *p*-value of <0.05 was considered statistically significant.

## 3. Results

### 3.1. Pretreatment with EWE and bEWE via Drinking Water Attenuated the Growth of Orthotopic Lung Carcinoma in Mice

The oral administration of water extracts from *E. gracilis* (EWE, bEWE, and PWE) via drinking water in mice was performed to evaluate their possible antitumor effects (Figure 1A). Body weights and water consumption were monitored every three days throughout the study period, and these were relatively consistent (Figures S1 and S2). The average body weights at the end of the study period were 20.6 ± 0.6, 20.6 ± 0.7, 20.6 ± 0.6, and 20.5 ± 0.7 g with PBS, EWE, bEWE, and PWE treatment, respectively. Water consumption was 3.8 ± 0.3, 4.2 ± 0.2, 4.4 ± 0.3, 4 ± 0.3 mL/mouse/day with PBS, EWE, bEWE, and PWE treatment, respectively. As shown in Figure 1B, tumor growth was attenuated in the group pretreated with an average of 202 ± 12 mg/kg/day EWE (tumor weight = 107.0 ± 103.0 mg, *p* > 0.05) as compared with the PBS control group (tumor weight = 203.2 ± 91.6 mg). Treatment with an average of 213.1 ± 13.3 mg/kg/day bEWE further attenuated the growth of LLC tumors in the lungs of the mice (tumor weight = 19.9 ± 31.3 mg, *p <* 0.05). However, this attenuation was not observed in the group treated with an average of 19.7 ± 2 mg/kg/day PWE (tumor weight = 152.8 ± 199 mg, *p* > 0.05). Although the dosage of the PWE is 1/10 of those of EWE or bEWE, this is reflective of the yield of PWE from the same amount of *Euglena gracilis*. As shown in Figure 1C, the EWE- and bEWE-treated mice developed fewer and smaller tumor nodules as compared to the PBS control group. Either EWE or bEWE treatment via drinking water did not alter mouse growth as measured by body weight (Figure S1). These results indicate that *Euglena* water extracts (EWE and bEWE) are effective in inhibiting the lung carcinoma growth in mice. A noteworthy finding in this study is that the bEWE is significantly more effective than the EWE in inhibiting lung tumor growth. This suggests that the heat treatment of EWE for 10 min in boiling water may be an important step in recovering the anticancer activity of *Euglena gracilis*.

### 3.2. Euglena Water Extracts Alter the Gut Microbiota Composition

*Euglena* water extracts (EWE and bEWE) reduce lung carcinoma growth in mice (Figure 1), which concurs with our recent study [19]. Since *Euglena* water extracts were administered daily via drinking water, there is a possibility of alteration of intestinal microbiota and/or their metabolisms. We, therefore, analyzed the gut microbiome using 16S rRNA sequencing with the Illumina MiSeq platform in feces from the following groups: PBS (control), mice treated with EWE, and mice treated with bEWE, at three different time points (Day 0: before any treatment, Day 21: before LLC cell injection, Day 42: after LLC cell injection).

**Figure 2:**
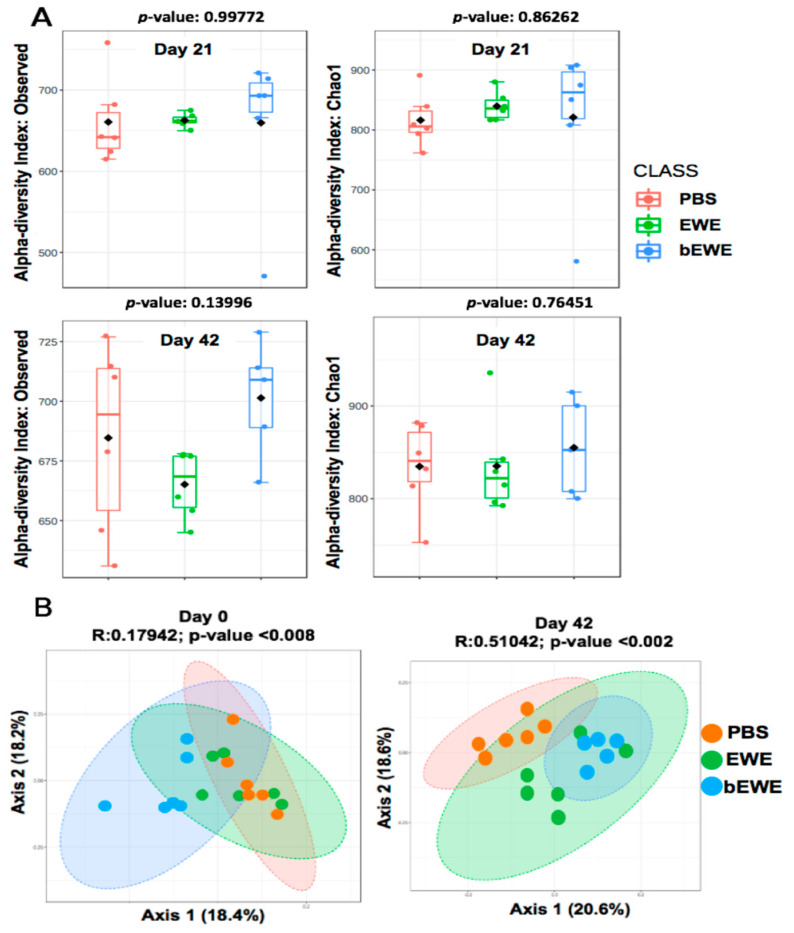
Alpha and beta diversity of gut microbiota in *Euglena* water extracts and PBS treated mouse feces before (Day 21) and after LLC cell inoculation (Day 42). (**A**) The difference in alpha diversity; measured as observed index and Chao1 index for Day 21 (upper panels in **panel A**) and Day 42 (lower panels in **panel A**). Box-and-whisker plots show high, low, and median values, with lower and upper edges of each box denoting first and third quartiles, respectively. (**B**) Principal coordinates analysis (PCoA) based on Bray-Curtis dissimilarity representing the difference in gut microbial communities. Each PCoA plot is accompanied by an analysis of similarity (ANOSIM) using an appropriate distance matrix. The R-values closer to zero indicates no difference between groups.

At a 97% similarity level, we obtained 25620 OTUs and 11 phyla, 53 families, 104 genera after statistical analysis of 16S rRNA sequences. Statistical analyses including alpha and beta diversity were quantified using observed index and Chao1 index and a principal coordinates analysis (PCoA), respectively. Chao1 estimates the total richness of the microbial community in a sample [36,37]. Our results showed no significant difference in alpha diversity between EWE- or bEWE-treated LLC tumor-bearing mice as compared to the PBS-treated group at both Day 21 and Day 42 (Figure 2A).

Beta diversity with principal coordinate analysis (PCoA) showed that bEWE- and EWE-treated mice had significantly different gut microbiome compositions (Figure 2B. ANOSIM, R = 0.51, *p* < 0.01) as compared with PBS treated mice at Day 42 (ANOSIM, R = 0.18, *p* < 0.01); however, some overlap between EWE and bEWE was observed, likely because both of these extracts were prepared from dried *Euglena* powder. ANOSIM based on Bray-Curtis distance indicated notable differences in microbial communities recovered from the fecal samples of LLC tumor-bearing mice treated with PBS, EWE, and bEWE.

### 3.3. Euglena Water Extracts Caused Alteration in the Gut Microbial Communities

**Figure 3:**
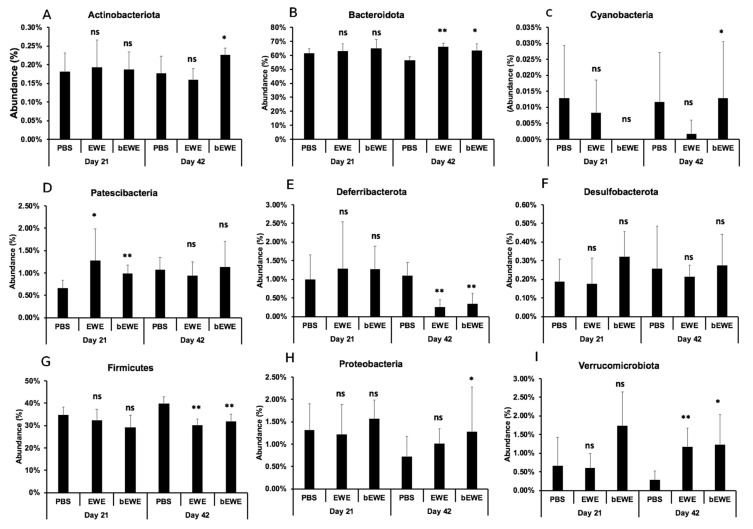
Relative abundance of bacterial taxa at the phylum level in mice treated with *Euglena* water extracts at Day 21 (before LLC cell inoculation) and Day 42 (after LLC cell inoculation). Percentage of taxa abundance of (**A**) Actinobacteriota, (**B**) Bacteroidota, (**C**) Cyanobacteria, (**D**) Patescibacteria, (**E**) Deferribacterota, (**F**) Desulfobacterota, (**G**) Firmicutes, (**H**) Proteobacteria, and (**I**) Verrucomicrobiota is presented. The difference in taxa abundance for each phylum between PBS and EWE or bEWE was compared at Day 21 and Day 42 using a *t*-test. Results are presented as mean ± SD (*n* = 5–6). *: *p* < 0.05; **: *p* < 0.01; ns: *p* > 0.05.

**Figure 4:**
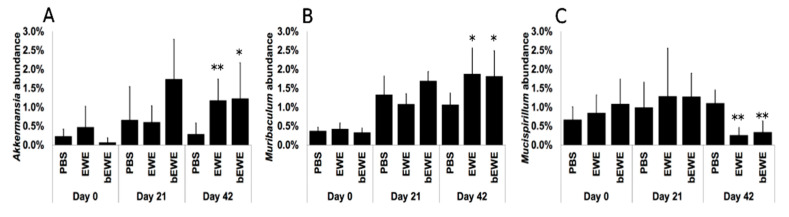
Relative abundance of bacterial taxa at the genus level in mice treated with *Euglena* water extracts at Day 21 (before LLC cell inoculation) and Day 42 (after LLC cell inoculation). Percentage of taxa abundance of (**A**) *Akkermansia*, (**B**) *Muribaculum*, and (**C**) *Mucispirillum* between PBS and EWE or bEWE were compared at Day 21 and Day 42 using *t*-test. Results are presented as mean ± SD (*n* = 5–6). *: *p* < 0.05; **: *p* < 0.01.

A taxon-dependent analysis was conducted to explore the gut flora phylum and genus that potentially mediate or are closely associated with the attenuation of lung carcinoma growth upon *Euglena* water extracts administration. At the phylum level, the analysis showed the Bacteroidota and Firmicutes were the most abundant phyla identified. We observed that Bacteroidota abundance was significantly higher but Firmicutes abundance was significantly lower in the groups treated with *Euglena* extracts at Day 42 (after LLC cell inoculation) than in the PBS-treated group (Figure 3B,G). Furthermore, bEWE treatment at Day 42 was associated with a significantly higher abundance of Actinobacteriota and Proteobacteria when compared to PBS treatment (Figure 3A,H). We also observed a significantly higher abundance of Verrucomicrobiota, but a significantly lower abundance of Deferribacterota, in groups treated with *Euglena* water extracts at Day 42, when compared to the PBS treated group (Figure 3I,E). Currently, *Akkermansia muciniphila* is the only identified representative species of the phylum Verrucomicrobiota [38], and *Akkermansia* abundance was also significantly higher with EWE or bEWE treatment (Figure 4A). Further, significantly higher abundance of *Muribaculum* (phylum Bacteroidota) and significant decrease of *Mucispirillum* (phylum Deferribacterota) was observed at Day 42 with EWE or bEWE treatment, rather than with PBS treatment (Figure 4B,C).

### 3.4. Transplantation of Fecal Microbiota from EWE-Treated Mice Resulted in Attenuation of Lung Cancer Growth

**Figure 5:**
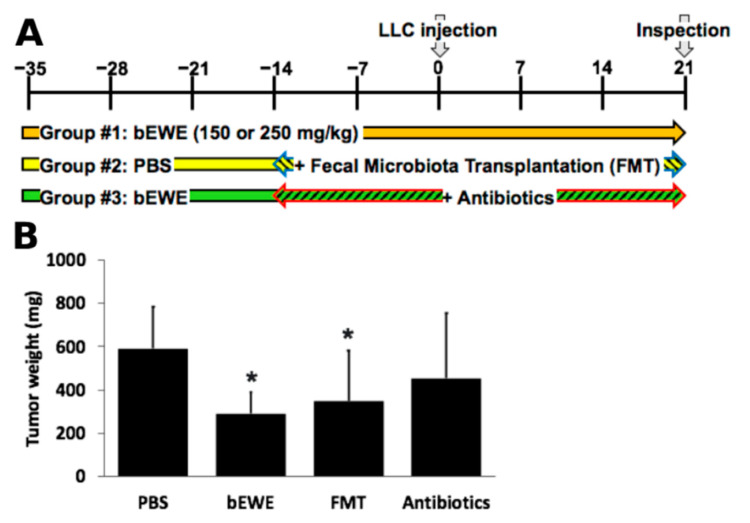
Fecal microbiota transplantation (FMT) from bEWE treated mice significantly attenuated the growth of tumors in the murine lung. (**A**) Schematic illustration of the study design. (**B**) Average tumor weight in each treatment group. PBS served as control. Results are presented as mean ± SD (*n* = 5). *: *p* < 0.05 as compared to PBS-treated group.

To further understand the role of microbiota in the prevention of lung carcinoma growth in mice by daily oral treatment with *Euglena* extract treated by boiling (bEWE) (Group #1 in Figure 5A), experiments with fecal microbiota transplantation (FMT) (Group #2 in Figure 5A) and antibiotics treatment (ABX) (Group #3 in Figure 5A) were performed as shown in Figure 5A. After 3 weeks of bEWE administration via drinking water, feces were collected and used for FMT for 5 weeks (Group #2 in Figure 5A). From this experiment, we observed that FMT-treatment significantly attenuated the growth of tumors in the mouse lung (349.3 ± 232.0 mg, *p* < 0.05) as compared with the PBS control group (591.2 ± 194.1 mg). The FMT-induced reduction of the lung tumor growth was similar to that of bEWE treated mice, which also experienced a significant decrease in the tumor burden (289.9 ± 98.1 mg, *p* < 0.05) in comparison to the burden of the PBS groups. On the contrary, the ABX treatment reversed the bEWE treatment-induced tumor growth attenuation and the tumor burden (452.7 ± 302.9 mg) was similar to that of the control group. Although a little variation in body weight was observed between FMT and ABX groups (administered through oral gavage) and PBS and bEWE groups (administered via drinking water, Figure S3), these variations were not statistically significant. These results strongly suggest that the intestinal microbiota plays a role in the attenuation of lung carcinoma growth in mice by oral administration of bEWE.

## 4. Discussion

Compounds present in organisms of the genus *Euglena* have considerable potential as pharmaceuticals and nutraceuticals. Since *Euglena gracilis* is easy to cultivate and has a faster growth rate than other Euglenophyceae species, it is widely used in basic research as a model organism [9]. Although several studies have been conducted to determine the therapeutic properties of substances obtained from *Euglena* against various diseases, including cancers [14,15,39,40], their effect on the gut microbiota is still not well understood. In the present study, we investigated the effect of oral daily administration of *E. gracilis* water extracts devoid of paramylons on the gut microbiota of mice and its association with the attenuation of lung cancer growth. Pretreatment with two types of *Euglena* water extracts (EWE and bEWE) via drinking water attenuated the growth of orthotopic lung carcinoma in syngeneic mice (Figure 1B). Among two types of extract, bEWE (EWE with boiling)-induced tumor growth attenuation was much stronger than that by EWE. This result indicates that heat treatment and boiling for 10 min altered either the quality or quantity of antitumor substances in the EWE. Although it is difficult to pinpoint the specific mechanism by which the heat treatment increased the antitumor effect of EWE, due to the unknown chemical nature of this antitumor substance it is conceivable that boiling may have resulted in increasing the solubility and diffusion rate of the bioactive molecules, which could lead to their increased absorption and efficiency. Alternatively, boiling and the subsequent centrifugation may have removed a potential inhibitor against antitumor substances in the EWE. Indeed, large denatured molecules have been removed by this process (data not shown). Nonetheless, this experiment warrants a follow-up study to identify the antitumor substance(s) present in *Euglena* extracts.

The mouse study revealed that FMT using stools from bEWE-fed mice attenuated lung carcinoma growth at the same level as in mice who were orally administered bEWE (Figure 5B). We observed that the administration of *Euglena* water extracts (bEWE and EWE) increased the ratio of Bacteroidetes to Firmicutes along with a significant increase in the genera *Akkermansia* and *Muribaculum* in lung tumor-bearing mice (Figure 3B,G and Figure 4). These results suggest that oral administration of partially purified water extracts from *E. gracilis* alters the intestinal microbiota and induces a significant suppression of lung carcinoma growth, presumably due to changes in the bacteria itself and/or their metabolites.

The association between gut microbiota and lung cancer has received increased appreciation after recent studies indicated the involvement of the gut–lung axis in the development of cancers by altering the immune system [41]. In the present study, we found no significant difference in the alpha diversity of gut microbiota in mice treated with *Euglena* water extracts in comparison to the PBS-treated mice. However, the beta diversity analysis showed a significant difference in the microbial composition (Figure 2). It has been reported that the gut microbial composition in healthy humans is significantly different from that in patients with cancers, including lung cancer [25,41,42]. Therefore, an alteration of the gut microbiota composition appears to be significantly influential on carcinogenesis in the lung.

In the current study, we observed significant alterations in the gut microbiota at Day 42 (end of the study period) after LLC cell injection in mice treated with *Euglena* water extracts (Figure 3). The alterations were characterized as a significant increase in the relative abundance of microbes of the phyla Bacteroidota, Verrucomicrobiota, Actinobacteriota, and Proteobacteria, while there was a significant reduction for the phyla Firmicutes and Deferribacterota (Figure 3). Since bacteria of the phyla Bacteroidota and Firmicutes are most abundant in the gut of humans and mice, any alteration in these bacteria potentially disrupts immunological homeostasis, thereby leading to various disease conditions. In support of this notion, an increase has been reported in the ratio of Firmicutes to Bacteroidota in patients with irritable bowel syndrome (IBS), autism, hypertension, chronic fatigue syndrome, and cancer patients, in comparison to healthy humans [43,44,45,46]. Similarly, another study found a higher ratio of Firmicutes to Bacteroidota in lung cancer patients than in healthy controls [47]. The current study found that administration of *Euglena* water extracts significantly decreased lung tumor growth and the ratio of Firmicutes to Bacteroidota. Therefore, it is suggested that daily oral administration of *Euglena* water extracts induces an attenuation of lung carcinoma growth, at least in part by the alteration of the gut microbial ratios of Firmicutes and Bacteroidota.

The current study also found that the abundance of organisms of phylum Deferribacteres and genus *Mucispirillum* was significantly lower in the LLC tumor-bearing mice treated with the *Euglena* water extracts at Day 42 (Figure 3E and Figure 4C). Deferribacteres are intestinal flora that play a role in iron metabolism and are associated with bowel iron balance [48]. Increased iron metabolism is associated with malignant transformation and cancer progression, as cancer cells are highly dependent on iron compared to normal cells [49,50]. Therefore, higher iron metabolism increases the risk of cancers and promotes tumor growth [48]. Although the underlying mechanisms are still not clear, imbalances in pulmonary iron levels are associated with the development of lung cancers [51]. Results in the current study suggest that *Euglena* water extract-induced growth attenuation of lung cancer is possibly associated also with the disruption of iron metabolism.

In our study, the abundance of genera *Akkermansia* and *Muribaculum* was found to be significantly higher in mice treated with *Euglena* water extracts than those treated with PBS at Day 42 (Figure 4). Although the study did not include the species-level identification within *Akkermansia* and *Muribaculum*, the significant increase in abundance for these genera maybe linked to the attenuation of lung carcinoma growth. Currently, *Akkermansia muciniphila* is the only representative species of the phylum Verrucomicrobiota, which typically reside in the large intestine of humans and other animals [38]. *A. muciniphila* has been reported as a beneficial microorganism due to its displayed role in increasing anti-inflammatory functions, improving host metabolism, and enhancing cancer treatment [52,53]. A human study revealed that *A. muciniphila* is less abundant in patients with lung cancer and supplementation of *A. muciniphila* improves the efficacy of immune checkpoint inhibitor (PD-1 blockade) therapy in lung, skin, and renal cancer treatments [54]. In addition, the present study also found that the abundance of *Muribaculum*, a genus belonging to the phylum Bacteroidota, was increased in the LLC tumor-bearing mice treated with *Euglena* water extracts (Figure 4B). Because an increase in *Muribaculum* is associated with better therapeutic efficacy of natural compounds in LLC tumor-bearing mice [55], it is suggested that *Akkermansia* and *Muribaculum* are the gut bacteria that could play a significant role in the *Euglena* water extracts-induced attenuation of lung carcinoma growth in mice.

## 5. Conclusions

In summary, the present study revealed that oral administration of *Euglena* water extracts (EWE and bEWE) prevents lung cancer growth in an orthotopic syngeneic mouse model. Daily oral administration of *Euglena* water extracts caused significant alteration in the gut microbiota, including an increase in the ratio of Bacteroidota to Firmicutes, an increase in Verrucomicrobiota, a decrease of Deferribacteres, and an increase of the genera *Akkermansia* and *Muribaculum*. The FMT and ABX studies confirmed that the attenuation of lung carcinoma growth in mice by *Euglena* water extracts is associated with the alteration of the gut microbiota. The alteration of the gut microbiome may induce microbial metabolite-dependent suppression in cancer growth inhibition. Future studies are needed to identify the bacterial metabolites involved in lung cancer growth attenuation and the mechanisms by which they attenuate the growth of lung cancer. The current study strongly suggests that oral daily administration of *Euglena* water extracts alters the gut microbiota, thereby attenuating lung carcinoma growth. This study indicates that the water extract from *E. gracilis* is a promising prebiotic for the prevention of lung cancer. This study also provides insights for developing a new rationale for lung cancer prevention strategy which would be of great value for human health.

## Data Availability

All data is available upon request to the corresponding author.

## Supplementary Materials

The following are available online at https://www.mdpi.com/article/10.3390/nu14030678/s1, Figure S1. Treatment of mice with water extracts from *E. gracilis* didn’t alter the body weight. Body weight was 20.6 ± 0.6, 20.6 ±0.7, 20.6 ± 0.6, and 20.5 ± 0.7 g in average with PBS, EWE, bEWE, and PWE treatments, respectively; Figure S2. Consumption of water with different treatments was relatively consistent throughout the study. Water consumption were 3.8 ± 0.3, 4.2 ± 0.2, 4.4 ± 0.3, 4 ± 0.3 mL/mouse/day with PBS, EWE, bEWE, and PWE treatments, respectively; Figure S3. The body weight of mice treated with PBS, bEWE, FMT, or antibiotics was comparable. The average body weights at the end of the study period were 19.9 ± 1.0, 19.8 ± 1.0, 18.9 ± 0.6, and 19.4 ± 0.6 g with PBS, bEWE, FMT, and bEWE + Antibiotics treatments, respectively.
