# Supplementary material for: Oral Administration of Water Extract from Euglena gracilis Alters the Intestinal Microbiota and Prevents Lung Carcinoma Growth in Mice
Source: Nutrients. 2022 Feb 5;14(3):678. doi: 10.3390/nu14030678 (PMC8839094; doi:10.3390/nu14030678)
Supplement: Supplementary file 1 [file nutrients-14-00678-s001.zip › nutrients-1550019-Supplementary Materials.pdf]

Supplementary Materials

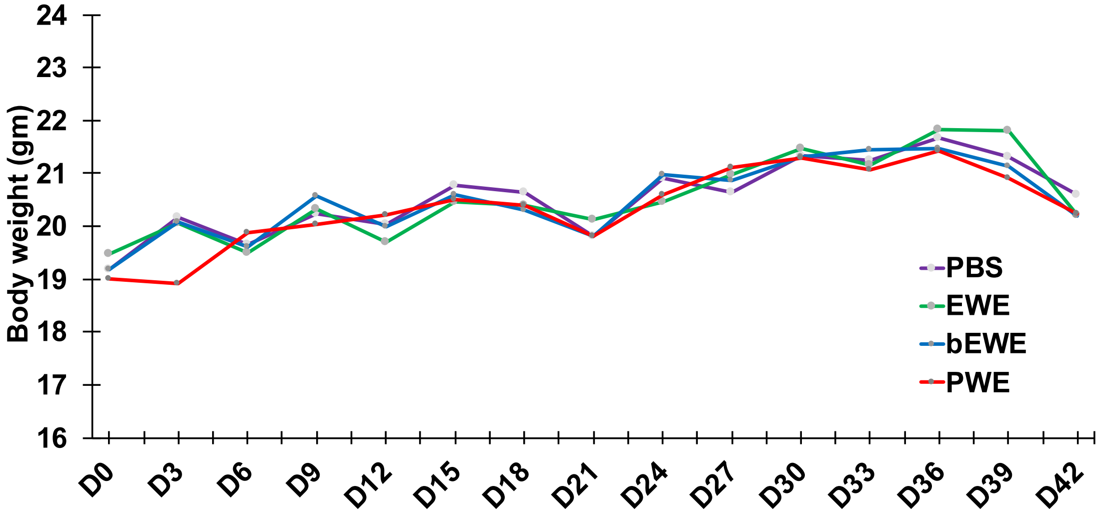

Figure S1. Treatment of mice with water extracts from *E. gracilis* didn't alter the body weight. Body weight were 20.6 ± 0.6, 20.6 ±0.7, 20.6 ± 0.6, and 20.5 ± 0.7 g in average with PBS, EWE, bEWE, and PWE treatments, respectively.

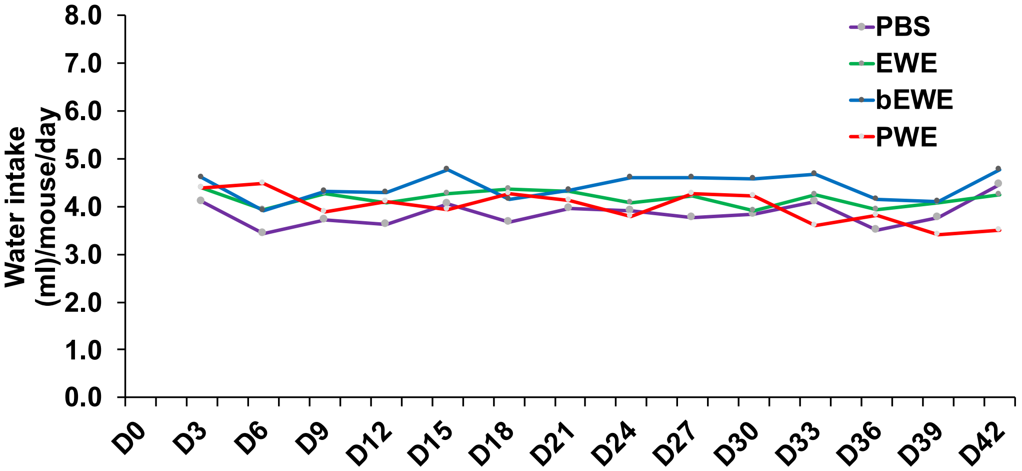

Figure S2. Consumption of water with different treatment was relatively consistent throughout the study. Water consumption were 3.8 ± 0.3, 4.2 ± 0.2, 4.4 ± 0.3, 4 ±0.3 ml/mouse/day with PBS, EWE, bEWE, and PWE treatments, respectively.

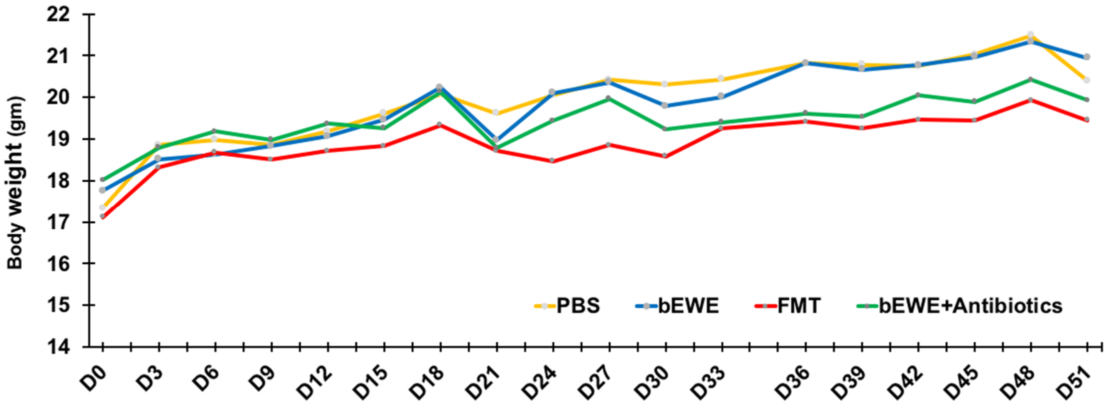

Figure S3. The body weight of mice treated with PBS, bEWE, FMT, or antibiotics was comparable. The average body weights at the end of the study period were 19.9 ± 1.0, 19.8 ± 1.0, 18.9 ± 0.6, and 19.4 ±0.6 g with PBS, bEWE, FMT, and bEWE + Antibiotics treatments, respectively.
